# Evaluation of Nonviral *piggyBac* and *lentiviral* Vector in Functions of CD19chimeric Antigen Receptor T Cells and Their Antitumor Activity for CD19^+^ Tumor Cells

**DOI:** 10.3389/fimmu.2021.802705

**Published:** 2022-01-10

**Authors:** Zhicai Lin, Xiangzhen Liu, Tao Liu, Haixia Gao, Sitong Wang, Xingli Zhu, Lijie Rong, Jingbo Cheng, Zhigang Cai, Fu Xu, Xue Tan, Linjie Lv, Zhong Li, Yan Sun, Qijun Qian

**Affiliations:** ^1^ Medical, Cell Product and R&D Department, Center for Cell Pharmaceuticals, Shanghai Cell Therapy Group, Shanghai, China; ^2^ R&D Department, Nucleotide Center, Shanghai Cell Therapy Group, Shanghai, China; ^3^ Department of Immunotherapy, Shanghai Cell Therapy Research Institute, Shanghai, China; ^4^ Shanghai Menchao Cancer Hospital, Shanghai University, Shanghai, China

**Keywords:** chimeric antigen receptor, CD19, B lymphoma, *piggyBac*, T cells, lentiviral

## Abstract

Nonviral transposon *piggyBac* (PB) and *lentiviral* (LV) vectors have been used to deliver chimeric antigen receptor (CAR) to T cells. To understand the differences in the effects of PB and LV on CAR T-cell functions, a CAR targeting CD19 was cloned into PB and LV vectors, and the resulting pbCAR and lvCAR were delivered to T cells to generate CD19pbCAR and CD19lvCAR T cells. Both CD19CAR T-cell types were strongly cytotoxic and secreted high IFN-γ levels when incubated with Raji cells. TNF-α increased in CD19pbCAR T cells, whereas IL-10 increased in CD19lvCAR T cells. CD19pbCAR and CD19lvCAR T cells showed similar strong anti-tumor activity in Raji cell-induced mouse models, slightly reducing mouse weight while enhancing mouse survival. High, but not low or moderate, concentrations of CD19pbCAR T cells significantly inhibited Raji cell-induced tumor growth *in vivo*. These CD19pbCAR T cells were distributed mostly in mesenteric lymph nodes, bone marrow of the femur, spleen, kidneys, and lungs, specifically accumulating at CD19-rich sites and CD19-positive tumors, with CAR copy number being increased on day 7. These results indicate that pbCAR has its specific activities and functions in pbCAR T cells, making it a valuable tool for CAR T-cell immunotherapy.

## Introduction

Chimeric antigen receptor (CAR) T-cell immunotherapy has shown promise in the treatment of hematologic malignancies. CD19-targeted CAR T cells were found to induce complete remission of disease in up to 90% of patients with relapsed or refractory B-cell acute lymphoblastic leukemia (B-ALL) who had an expected complete response rate to chemotherapy of 30% (1). However, due to the stringent requirements in the preparation of CD19CAR T cells, the estimated cost of treatment with Zuma-1 ranged from $896,600 to $1,615,000 per quality-adjusted life-year gained (2), and each treatment with Tisagenlecleucel was estimated to cost $475,000 (3). To reduce costs, a more economic approach is needed to produce CAR T cells.

The gene delivery tool is key to the generation of CAR T cells, with viral and nonviral vectors frequently utilized for gene delivery. Viral vectors include adenoviral (AV), adeno-associated viral (AAV), and lentiviral (LV) vectors. AV vectors have immunogenic properties when directly administered *in vivo*, whereas LV vectors require large-scale production by living cells (4). Nonviral delivery systems include cationic lipids and cationic polymers (5), as well as nonviral plasmids. Transposons are natural vectors for gene delivery (6). These vectors, which carry a gene expression unit called a “transgene,” can overcome some of the limitations of viral vectors (4). Three transposons have been described to date, *sleeping beauty* (SB), *Tol2*, and *piggyback* (PB). PB and SB have high transposition activities in mammalian cells, with PB having stronger activity (7) and encompassing larger chromatin loops (8) than SB. Because it leaves no footprint after excision, PB is less likely to cause genomic damage during mutagenesis (9). The use of PB transposition to express a CAR resulted in the generation of CAR T cells targeting CD19, which can be used to treat patients with B-lineage malignancies and acute lymphoblastic leukemia (10, 11).

CAR T cells could be economically developed by generating a *piggyBac* transposase through an established one-plasmid screening system and a two-step high-throughput screening process. The most hyperactive transposase, bz-hyPBase, with four mutation sites showed high-efficiency editing ability in Chinese hamster ovarian carcinoma (CHO) cells and T cells (12). Similarly, *piggyBac* transposons have been used to develop CAR T cells for mesothelin (13–15), EGFR (16), and glypican-3 (17). This study evaluated the abilities of pbCAR and lvCAR to generate CD19CAR T cells and the activities of these cells *in vitro* and *in vivo*. Both formulations of CD19CAR T cells increased interferon-γ (IFN-γ) levels when incubated with Raji cells. tumor necrosis factor-α (TNF-α) was increased in CD19pbCAR T cells, whereas IL-10 was increased in CD19lvCAR T cells. High doses of CD19pbCAR T cells showed strong cytotoxic activity against Raji cells *in vitro* and Raji cell-induced tumors *in vivo*, and mice treated with CD19pbCAR T cells survived longer and at high rates. These results demonstrated that pbCAR may become an effective tool for generating CAR T cells that can be used in cancer immunotherapy.

## Materials and Methods

### Cell Lines

CD19^+^ Raji cells were purchased from the American Type Culture Collection (ATCC, Shanghai, China). CD19^KO^ Raji cells were generated from Raji cells by CRISPR/Cas9-mediated knockout of CD19 through GeneScript. Luciferase-expressing Raji cells (Raji-Luc-C8) were purchased from Beijing Idmo Co., Ltd. (Beijing, China). All of these cells were cultured in RPMI-1640 medium containing 10% fetal calf serum at 37°C in an atmosphere containing 5% CO_2_.

### Generation and Expansion of CD19pbCAR T Cells or CD19pbCAR T-luc Cells and CD19lvCAR T Cells or CD19lvCAR T-luc Cells

**Figure 1:**
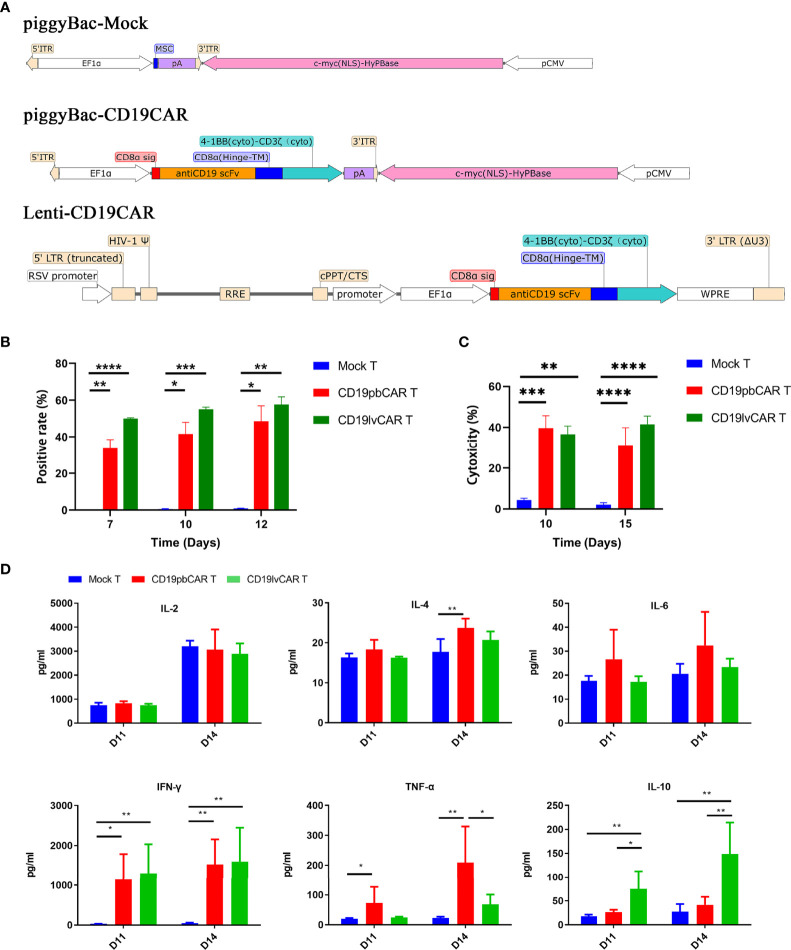
Generation and characterization of CD19CAR T cells with different vectors. **(A)** Construction maps of *piggyBac* (PB) Mock, *piggyBac*-CD19CAR, and *lentivirus* (lv)-CD19CAR. **(B)** Rates of CAR T-cell positivity for vectors over time. CAR T cells were electroporated with *piggyBac* Mock or *piggyBac*-CD19CAR or infected with lv-CD19CAR. **(C)** Cytotoxicity of Mock T, CD19pbCAR T, and CD19lvCAR T cells against CD19^+^ Raji cells at days 10 and 15. **(D)** Cytokine levels in the medium after incubation of Mock T, CD19pbCAR T, and CD19lvCAR T cells with 1 × 10^5^ Raji cells for 4 h. *p<0.05; **p<0.01; ***p<0.001; ****p<0.0001.

DNA sequences encoding a second-generation CAR containing an FMC63-derived scFv domain for CD19 and a 4-1BB costimulation domain were cloned into pNB338B-EF1α vector with c-myc (nuclear localization signal (NLS)) containing the PB transposon, yielding CD19pbCAR, and into lentiviral vector, yielding CD19lvCAR. *PiggyBac* vector alone was used as control yielding Mock T (**Figure 1A**). All plasmids were verified by sequencing before transfection.

Peripheral blood mononuclear cells (PBMCs) from whole blood were purchased from AllCells (Shanghai, China) and isolated by Ficoll density gradient centrifugation (Cat#17-5442-03, GE). A total of 1 × 10^7^ PBMCs in reaction buffer were electroporated with 6 μg Mock plasmid, CD19pbCAR, or CD19pbCAR T-luc using a Lonza/VPA-1002 device. The cells were gently and immediately transferred to 6-well plates with prewarmed AIM-V medium (Cat# A3021002, Life Technologies, Carlsbad, CA, USA) and incubated for 4 h. The cells were subsequently transferred to plates coated with standard cell stimulation solution containing anti-CD3 (clone 145-2C11, BD Pharmingen, San Diego, CA, USA) or CD19 (Cat# CD9-H5259, Acro, Newark, DE, USA) and anti-CD28 mAb (Cat#A028H, Shanghai Cell Therapy Group Corp., Shanghai, China) in AIM-V medium supplemented with interleukin-2 (IL-2; Cat# S20020004, Shandong Quangang Pharmaceutical Co., Jinan, China) and CTS™ immune cell serum replacement (Cat#A2596101, Life Technologies) and incubated for 5 days. The activated cells were expanded in AIM-V medium with 100 UI IL-2 until day 13. Aliquots of cells were withdrawn every 2 days and counted using a Cellometer K2 cell counter (Nexcelom Bioscience, Lawrence, MA, USA). CD19pbCAR T cells were harvested and cryopreserved for later characterization and functional analysis.

CD19lvCAR and CD19lvCAR-luc were designed in our laboratory; their proliferation and purification were performed by Shanghai Genechem Company (Shanghai, China). T cells were infected with the lentiviral CARs according to the manufacturer’s instructions. The infected cells were cultured under the same condition as CD19pbCAR T cells.

### Cytotoxicity Assay

CAR-mediated cytotoxicity was tested using DELFIA EuTDA cell cytotoxicity assays. Briefly, 1 × 10^5^ Raji cells were labeled with BATDA for 30 min at 37°C, followed by washing three times with PBS to remove excess BATDA. CD19pbCAR T cells and CD19lvCAR T cells were added to the BATDA-labeled Raji cells in 96-well V-bottom plates at effector/target (E:T) cell ratios of 16:1, 8:1, 4:1, 2:1, and 1:1, and the plates were incubated at 37°C for 4 h in an atmosphere containing 5% CO_2_. Europium solution was added, and the supernatants were harvested and their absorbance was measured using an automatic microplate reader (Perkin Elmer Envision). Percent-specific lysis was calculated as (readout − spontaneous release)/(maximum release − spontaneous release) × 100.

### Cytokine Assays

The concentrations of cytokines released by CD19pbCAR T cells were measured using human Th1/Th2 cytokine II assay kits. CD19pbCAR T cells were thawed, washed with AIM-V medium, and cocultured with Raji cells at E:T ratios of 16:1 to 1:1 for 24 h. A 100-µl aliquot of supernatant was withdrawn for cytokine detection by flow cytometry (Beckman Coulter, Brea, CA, USA). The cytokines IL-2, IFN-γ, TNF-α, IL-4, IL-6, and IL-10 were measured with corresponding antibodies and analyzed with FCAP Array v3.0 software.

### Analysis of Phenotype of CAR T Cells

The phenotypes of CAR T cells were analyzed during expansion. CAR+ cells were incubated with CD19-biotin (GeneScript, Piscataway, NJ, USA) followed by PE-avidin. Cytotoxic CD8+ T cells and helper CD4+ T cells were measured using FITC-conjugated antihuman CD4 and PE-conjugated antihuman CD8 antibodies. Central memory T (Tcm) cells were measured using APC-conjugated antihuman CD45RO and FITC-conjugated antihuman CD197 (CCR7) antibodies. Exhausted T cells were measured using PE-conjugated antihuman CD279 (PD-1), Brilliant Violet 510™-conjugated antihuman CD223 (LAG3), and APC/cyanine7-conjugated antihuman CD366 (Tim-3) antibodies. Samples were assayed on a Navios™ flow cytometer (Beckman Coulter, Brea, CA, USA) and analyzed using Kaluza analysis software. All of the above antibodies were purchased from BioLegend or BD Bioscience.

### *In Vivo* Evaluation of CD19pbCAR T Cells in a Mouse Model

1) To compare the effects of CD19pbCAR T cells and CD19lvCAR T cells against Raji cell-induced tumors, 1.0 × 10^6^ Raji-luc cells were injected into the tail veins of 6- to 8-week-old female NPI (NOD-Prkdc^scid^-il2rg^em1IDMO^) mice (Beijing IDMO Co., Beijing, China), which were maintained in standard sterile rooms with daily monitoring. The mice were randomly divided into three groups of six mice each and were injected intravenously with 2 × 10^7^ CD19pbCAR T cells, CD19lvCAR T cells, or PBS (control group). Tumor developed was assessed weekly by bioluminescence imaging using the Caliper IVIS-Lumina-XR (**Figure 3**).

2) The *in vivo* efficacy of CD19pbCAR T cells was assessed in a Raji-Luc-C8 xenograft model, which was established by intravenously injecting 1 × 10^6^ Raji-Luc-C8 cells into NPI mice. Ten days after injection, the 40 mice were randomly divided into five groups of eight mice each. These groups were intravenously injected with vehicle, 2.5 × 10^6^ Mock T cells, 2.5 × 10^6^ CD19pbCAR T cells (low dose), 5.0 × 10^6^ CD19pbCAR T cells (middle dose), or 10 × 10^6^ CD19pbCAR T cells (high dose). Bioluminescence was assessed using a Xenogen IVIS Spectrum System (PerkinElmer, USA) (Life Technologies, USA) on days 0, 3, 7, 14, 28, 42, 50, and 61 (**Figure 5A**).

3) To measure tumor volume, Raji cells were injected subcutaneously into 24 NPI mice. The mice were randomly divided into three groups, followed by injection into the tail vein of vehicle, 2.5 × 10^6^ Mock T cells and 10 × 10^6^ CD19CAR T cells. Tumor length and width were measured every 3 days with Vernier calipers for 24 days, and tumor volume were calculated as length × width 2/2 (**Figure 5D**).

4) The distribution of CD19pbCAR T cells was assessed in NPI (NOD-Prkdc^scid^-il2rg^em1IDMO^) mice (Shanghai Biocytogen Company). A single dose of 1 × 10^6^ Raji-Luci-C8 cells was administrated to each of 21 mice, followed up 9 days later by intravenous injection of 1 × 10^7^ CD19pbCAR T cells. Three mice each were sacrificed by isoflurane administration on days 1, 3, 7, 10, 15, 22, and 29. Samples of the brain, liver, spleen, heart, lungs, kidneys, mesenteric lymph nodes, bone marrow of the femur and sternum, stomach, duodenum, pancreas, uterus, and ovaries were collected, and DNA was extracted using DNeasy Blood & Tissue Kits (Qiagen, Hilden, Germany). CD19pbCAR T cell distribution in these tissues were assessed by quantitative PCR using primers and probes for CD19pbCAR and β-actin designed and synthesized by Sangon Biotech (Shanghai, China) (**Figures 6A, B** **)**.

To test selective binding of CD19^+^ cells, 1 × 10^7^ Raji cells were subcutaneously injected into right low backs of NOD-prkdSCID IL-2Rγ^−/−^ mice (B-NSG, Beijing Biocytogen Co., Ltd). When the tumors had grown to about 200 mm^3^ in size, the mice were administered 2 × 10^7^ CD19pbCAR T-luc cells or Mock T-luc cells *via* the tail vein. Fluorescent signals of CD19pbCAR T-luc cells were monitored on days 1, 7, 14, and 20 using IVIS-Lumina-XR calipers (**Figures 6C, D** **)**.

To evaluate pharmacodynamics and toxicity, clinical symptoms were observed every day, whereas body weights and fluorescence intensities were measured as described. All mice were sacrificed at 40 weeks.

### Statistical Analysis

All data are presented as mean ± SD. Differences between two independent groups were evaluated by Student’s *t*-tests, whereas difference among three or more groups was evaluated by one-way ANOVA. Survival was analyzed by the Kaplan-Meier method and compared by log-rank tests. All statistical analyses were performed using GraphPad Prism 6.0, with *p* < 0.05 considered statistically significant.

## Results

### Construction of CD19pbCAR and CD19lvCAR and Expansion of CAR T Cells

DNA sequences encoding a full single-chain variable fragment (scFv) for CD19 was fused with a CD8α hinge, a 4-1BB transmembrane domain, and a CD3ζ intracellular signaling domain and inserted into a *piggyback* transposon or a lentivirus (**Figure 1A**). The resulting pbCAR was amplified in *Escherichia coli* and purified with a plasmid endotoxin-free kit. Lentiviral CAR was prepared by the Shanghai Genechem Company. Human lymphocytes were isolated from the peripheral blood of normal donors and expanded as described (15). Briefly, 6 µg of CD19pbCAR or MOCK vector were transfected into 1 × 10^6^ T cells by electroporation (Lonza, Basel, Switzerland). Alternatively, T cells were infected with CD19lvCAR at a MOI of 10. The cells were incubated in AIM-V medium (GIBCO, Waltham, MA, USA) containing anti-CD3 and anti-CD28 antibodies and IL-2. Measurement of the percentages of CAR^+^ cells after 3 days showed that 30%–50% of CD19pbCAR T cells and 50%–58% of CD19lvCAR T cells were positive for CAR (**Figure 1B**).

### Activities and Phenotypes of CD19pbCAR T Cells and CD19lvCAR T Cells

Compared with Mock T cells, both CD19pbCAR T cells and CD19lvCAR T cells were highly cytotoxic (**Figure 1C**). The activities of two vectors were analyzed by measuring the cytokine concentrations of Mock T cells, CD19pbCAR T cells, and CD19lvCAR T cells 11 and 14 days after their incubation with 1 × 10^5^ Raji cells for 4 h. Compared with Mock T cells, both types of CAR T cells had significantly higher levels of IFN-γ (**Figure 1D**). CD19pbCAR T cells showed increased levels of IL-4 on day 14 and TNF-α on days 11 and 14, whereas CD19lvCAR T cells showed increased levels of IL-10 on days 11 and 14 (**Figure 1D**). Interestingly, levels of IL-2 and IL-6 were not significantly altered (**Figure 1D**).

**Figure 2:**
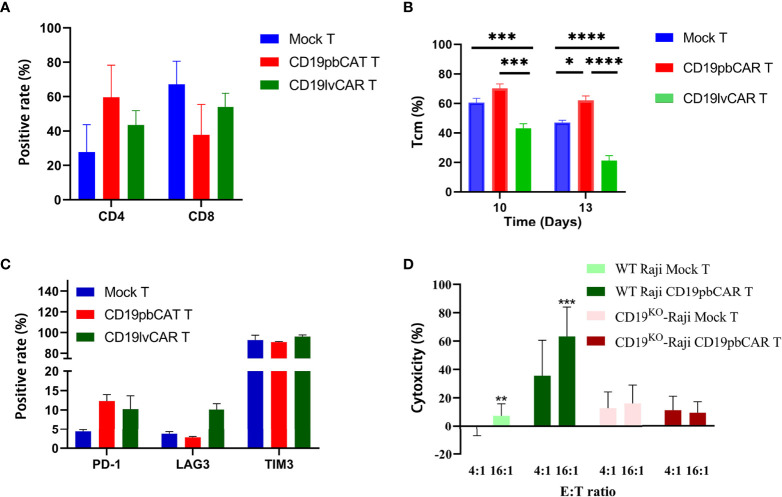
Phenotypes and specificities of CD19pbCAR T cells and CD19lvCAR T cells. **(A)** Proportions of CD4 and CD8 T cells measured on day 13 by flow cytometry. **(B)** Proportions of Tcm cells measured on days 10 and 13 by flow cytometry. **(C)** Expression of exhaustion markers (PD-1, LAG3, and TIM3) by flow cytometry on day 13. **(D)** Specificity of CD19pbCAR T cells, as shown by incubation with CD19^KO^ Raji cells. *p<0.05; **p<0.01; ***p<0.001; ****p<0.0001.

Flow cytometry analysis of cell phenotypes showed that the numbers of CD4^+^CAR^+^T cells were increased while the levels of CD8^+^CAR^+^T cells were decreased in both preparations of CAR T cells (**Figure 2A**). However, Tcm levels in CD19pbCAR T were increased (**Figure 2B**). The number of Tcm is an important marker in the evaluation of CAR T cell functions. Evaluation of the expression of exhaustion markers showed that PD-1 expression was increased in both preparations of CAR T cells, whereas LAG3 expression was increased only in CD19lvCAR T cells. TIM3 levels did not change in either preparation of CAR T cells (**Figure 2C**).

To determine if CD19pbCAR T could specifically target CD19 on cell surfaces, its effects on Raji cells in which the CD19 gene had been knocked out by CRISPR/Cas9 was tested. Although CD19pbCAR T cells killed CD19^+^ WT Raji cells at E:T ratios of 4:1 and 16:1, CD19pbCAR T cells were not cytotoxic to CD19^KO^ Raji cells at any E:T ratio (**Figure 2D**). The results demonstrate that the scFv used by CD19pbCAR T cells to target CD19 is specific and effective.

### Comparison of CD19pbCAR T Cells with CD19lvCAR T Cells in Treatment of Raji Cell-Induced Tumors in a Xenograft Model

**Figure 3:**
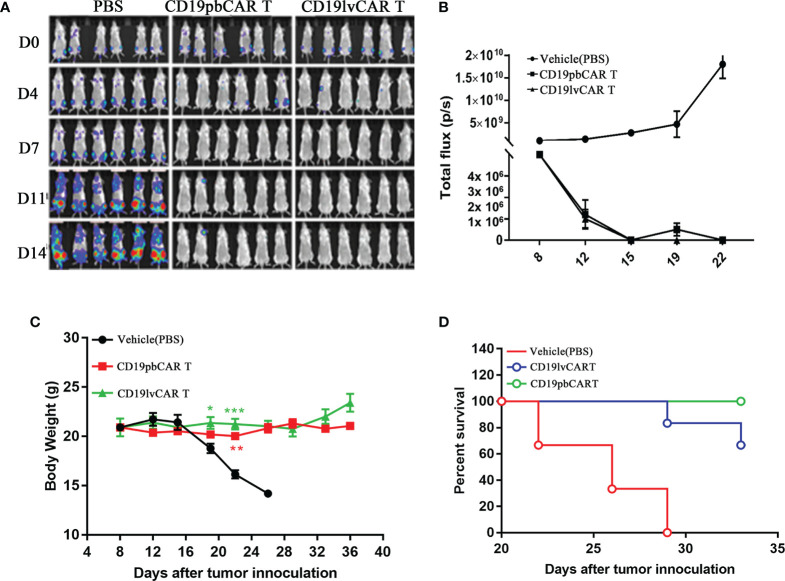
Comparison effects of CD19pbCAR T cells and CD19lvCAR T cells against Raji cell-induced tumors in a xenograft model. **(A)** Luciferase imaging, showing systemic trafficking and tumor accumulation of Raji cells. 1 x 10^6^ Raji-luc-C8 cells were injected into the tail veins of NPI mice, followed by the injection of the same volumes of PBS (vehicle), 1×10^7^ CD19pbCAR T cells and 1×10^7^ CD19lvCAR T cells. Fluorescence in each mouse was evaluated by the Caliper IVIS-lumina-XR on days 0, 4, 7, 11, and 14. **(B)** Measurements of tumor volumes at the indicated times. **(C)** Body weights of mice measured at the indicated times. **(D)** Numbers of surviving and dead mice determined until day 40. *p<0.05; **p<0.01; ***p<0.001.

To determine the efficiency of the pb and lv vectors in the preparation of CAR T cells, the tumoricidal effects of CD19pbCAR T cells and CD19lvCAR T cells in an *in vivo* xenograft mouse model were tested. Raji cells were injected into mouse tail veins, followed by administration of 1 ml PBS, 2 × 10^7^ CD19pbCAR T cells of 2 × 10^7^ CD19lvCAR T cells. Raji cells induced tumors in many tissues in the PBS group but these tumors were strongly inhibited by administration of CD19pbCAR T cells and CD19lvCAR T cells (**Figure 3A, B** **)**. Neither of these CAR T cell preparations affected mouse body weight during treatment (**Figure 3C**), whereas more mice treated with CD19pbCAR T cells than with CD19lvCAR T cells survived (**Figure 3D**).

### Proliferation and Activities of CD19pbCAR T Cells

**Figure 4:**
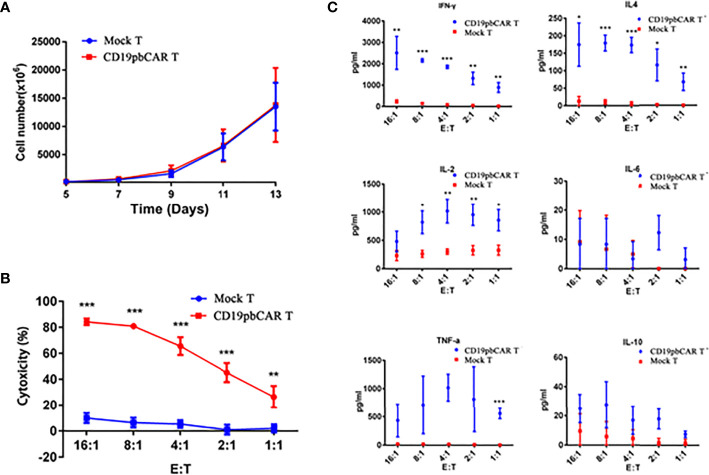
Proliferation and activities of CD19pbCAR T cells. **(A)** Proliferation of CD19pbCAR T cells 5-13 days after electroporation. **(B)** Cytotoxicity of Mock-T and CD19pbCAR T cells against Raji cells at different E:T ratios. **(C)** Cytokine levels of Mock-T and CD19pbCAR T cells 13 days after incubation with 1×105 Raji cells for 4 hours at different E:T ratios. *p<0.05; **p<0.01; ***p<0.001.

To determine the activities of CD19pbCAR T cells, the numbers of cells were measured from day 5 to day 14 after electroporation or infection with CAR vectors. Both Mock T and CAR T cells proliferated gradually, with no difference in growth rates (**Figure 4A**). To test their cytotoxicity, CD19pbCAR T cells were incubated with Raji cells at E/T ratios of 1:1, 2:1, 4:1, 8:1, and 16:1 for 24 h, and OD values were measured by Synergy H1 Hybrid Reader (BioTek, Bad Friedrichshall, Germany). CD19pbCAR T cells were found to have a stronger cytotoxic effect on CD19^+^ Raji cells than Mock T cells (**Figure 4B**).

Cytokines in the medium were also detected by flow cytometry. At all cell ratios, the levels of IFN-γ, IL-4, IL-2, and TNF-α were higher with CD19pbCAR T cells than with Mock T cells (**Figure 4C**). Compared with Mock T cells, CD19pbCAR T cells increased IL-2 levels at low E/T ratios but not at the highest ratio (16:1). TNF-α had pattern similar to IL-2. Interestingly, CD19pbCAR T cells did not increase the level of IL-6 except at a 2:1 ratio, whereas IL-10 levels were slightly higher with CD19pbCAR T cells than with Mock T cells. These findings suggested that CD19pbCAR T cells were more cytotoxic and had fewer effects.

### High Dose of CD19pbCAR T Cells Suppressed the Growth of Raji Cells in a Murine Hematological Malignancy Model

**Figure 5:**
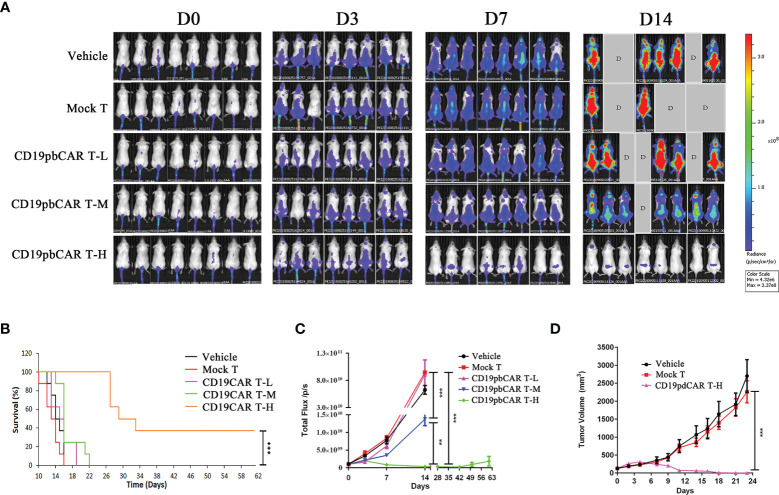
High concentration of CD19pbCAR T cells eliminated the growth of Raji cells in a murine model of hematological malignancy. **(A)** Luciferase imaging, showing systemic trafficking and tumor accumulation of Raji cells; 1 × 10^6^ Raji-luc-C8 cells were injected into the tail veins of NPI mice 10 days, followed by the injection of medium (vehicle), Mock T cells, or 2.5 × 10^6^ (low dose), 5.0 × 10^6^ (middle dose), or 10 × 10^6^ (high dose) CD19pbCAR T cells. Fluorescence in each mouse was evaluated by the Caliper IVIS-lumina-XR on days 0, 3, 7, and 14. **(B)** Numbers of surviving mice in the five groups. **(C)** Measurement of total fluorescence density of Raji tumor cells in each mouse at the indicated times. **(D)** Change in tumor volume in mice administered high dose CD19pbCAR T cells. **p<0.01; ***p<0.001.

NPI mice at age 6 weeks were injected in the tail veins with 1 × 10^6^ Raji-Luc-C8 cells. Ten days later, mice with tumors were injected with 2.5 × 10^6^ (low dose), 5.0 × 10^6^ (middle dose), or 10 × 10^6^ (high dose) CD19pbCAR T cells, or with vehicle or 2.5 × 10^6^ Mock T cells. Assessment of fluorescent signals from the Raji-Luc-C8 cells showed that these cells grew cells continually in the vehicle and Mock T cell groups but were inhibited in mice injected with low- and middle-dose CD19pbCAR T cells (**Figure 5A**). Some of the mice in the vehicle, Mock T cell, low- and middle-dose CD19pbCAR T-cell groups died by day 14, whereas none of the mice in the high-dose CD19pbCAR T-cell groups died. Except for mice in the high-dose CD19pbCAR T-cell group, all of the mice in other groups died by day 28 (**Figure 5B**). Injection of a high dose of CD19pbCAR T cells significantly eliminated tumor cells, showing very low flux reads (**Figure 5C**). Measurements of tumor volumes showed that tumors in the high-dose CD19pbCAR T-cell group were much smaller than tumors in the other groups, indicating that CD19pbCAR T cells can effectively eliminate CD19^+^ tumor cells (**Figure 5D**).

### CD19pbCAR T Cell Distribution in Mice and Their Specific Targeting of Subcutaneous CD19 ^+^ Tumor

**Figure 6:**
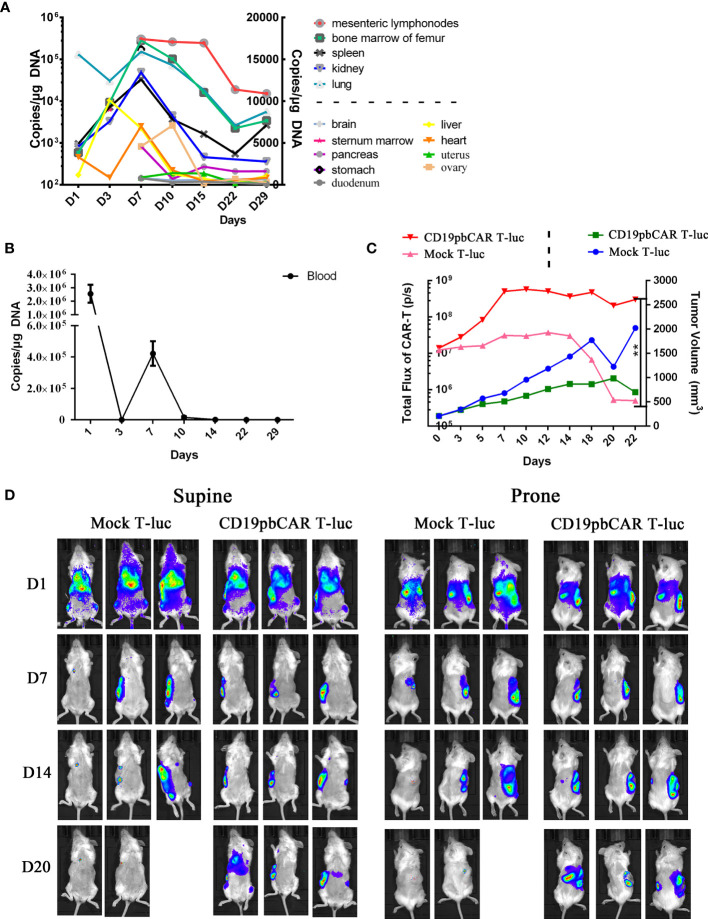
Distribution and specific accumulation of CD19pbCAR T cells. **(A)** Copies of pbCAR in tissues; 1 × 10^6^ Raji tumor cells were injected into the tail veins of 21 NPI mice, followed 9 days later by intravenous injection of 1 ×10^7^ CD19pbCAR T cells. Three mice each were euthanized on days 1, 3, 7, 10, 14, 22, and 29 for collection of tissues and blood. **(B)** Copies of pbCAR in the blood of the mice, as determined by qPCR, at the indicated times. **(C)** CD19pbCAR T cells specifically target transplanted tumor; 1 × 10^6^ Raji tumor cells were injected subcutaneously into the right back sides of mice. When tumor volume reached 200 mm^3^, the mice were injected with 2x10^7^ Mock T-luc or CD19pbCAR T-luc cells. Fluorescent images were taken at the indicated times. **(D)** Measurements of total body fluorescence and tumor volume. **p<0.01.

To determine the distribution and specific effect of CD19pbCAR T cells in mouse tissues, CD19pbCAR T cells were injected into NPI mice. Their tissues were collected at the indicated times, and the number of copies of CD19CAR in these tissues was analyzed by qPCR (**Figure 6A**). Higher concentrations of CAR T cells were observed in mesenteric lymph nodes, bone marrow of the femur, spleen, kidney, and lungs than in the brain, pancreas, stomach, liver, heart, uterus, ovaries, and duodenum (**Figure 6A**). Concentrations of CD19pbCAR T-luc cells showed two peaks on days 1 and 7 (**Figure 6B**). To assess the specific translocation of CD19pbCAR T cells, 1 × 10^7^ Raji cells were subcutaneously injected into the right side of mouse abdomens. When tumor nodule volumes reached about 200 mm^3^, 2 × 10^7^ of CD19pbCAR T-luc or Mock T-luc cells were injected into mouse tail veins, and bioluminescence, reflecting the distribution of CD19pbCAR T cells and Mock T cells, was evaluated on days 1, 7, 14, and 20 (**Figure 6C**). Both Mock T cells and CD19pbCAR T cells were distributed in most tissues over the first 24 h after injection. On days 7 and 14, fluorescence was observed on the right side in the supine position and on the left side in the prone position. After 20 days, fluorescence was observed only at the sites of Raji cell injection, suggesting that CD19pbCAR T cells selectively migrate to the locations of CD19^+^ Raji cells.

## Discussion

Human T cells with CD19CAR delivered by PB transposon demonstrated antitumor activity against CD19^+^ cells both *in vitro* and *in vivo*. These CD19pbCAR T cells strongly inhibited tumor cells in an animal model. The CD19pbCAR T cells were initially distributed widely throughout the body of these mice but migrated over time to the tumor site. Evaluation of CAR copy numbers showed two peaks after infusion, the first one day 1 after infusion and the second peak on day 7, suggesting the expansion of these CD19pbCAR T cells *in vivo*. Clinically, the injection of CD19pbCAR T cells would likely eliminate CD19^+^ tumor cells in the treatment of patients with B-cell malignancies.

Tcm cells show superior persistence and antitumor immunity in T-cell therapy, making them essential for treatment efficacy (18, 19). The present study found that the numbers of Tcm cells was greater in preparations of CD19pbCAR-transduced than of CD19lvCAR-infected T cells. The concentrations of IFN-γ, IL-4, and TNF-α were higher in CD19pbCAR T cells, suggesting that these cytokines were responsible for cytotoxicity. IFN-γ is required for cytotoxicity, whereas IL-4 is important for CAR T-cell expansion.

Cytokine releasing syndrome (CRS) is a major concern when CAR T cells are infused in cancer immunotherapy. IL-6 plays a critical role in the CRS. Interestingly, the concentrations of IL-6 and of the inhibitory cytokine IL-10 were not affected by CD19pbCAR, regardless of the E:T ratio. These findings suggest that CD19pbCAR T cells may have a relatively weak CRS *in vivo*.

Distribution analysis showed that injected CD19pbCAR T cells concentrate in the lymph nodes, bone marrow, spleen, kidneys, and lungs. CAR T cells spread most into hematologic tissues, where they may help eliminate the malignancies of the hematologic system. This study did not compare the distributions of CD19pbCAR T cells with CD19lvCAR T cells because previous studies have evaluated the distribution of lentiviral vectors for T-cell engineering (20). Better understanding of the properties of CD19pbCAR T may enable the design of more economical cellular medicines for cancer therapy.

The PB transposon (21) is active only when cotransfected with a PB transposase expression vector. Acting together, the transposon and transposase can integrate a gene into the genome of cells through a “cut and paste” mechanism. The PB transposon and transposase are introduced into T cells by electroporation, allowing them to stably express CD19-specific CAR (10) or the 4-D nucleofector system (11). Lentiviral delivery of CAR into T cells has been utilized in many clinical trials and cell drug products. Although lentiviral infection is more efficient than *piggyBac* transduction by electroporation, the *piggyBac* system generally uses TTAA as integration target sites. In addition, the *piggyBac* system does not require DNA synthesis during the actual transposition event and can maintain CAR expression for a longer period of time (22, 23).

The PB transposon has also been used to generate other CAR T cells, including anti-HER2 CAR T cells to treat HER2-positive tumors (24) and antigranulocyte-macrophage colony-stimulating factor receptor (GMR, CD116) CAR T cells to treat juvenile myelomonocytic leukemia (JMML) (25). This system has been shown to be efficient in the genetic modification of human T cells (26) and shows a lack of preference for integration into or near known proto-oncogenes (27). To improve the therapeutic efficacy of CAR T cells in an immunosuppressive tumor microenvironment, plasmids encoding CRISPR/Cas9 to disrupt the PD-1 gene and the *piggyBac* transposon to express CD133-specific CAR were cotransfected into human primary T cells. The resulting PD-1-deficient CD133-specific CAR T cells showed greater proliferation and cytotoxicity *in vitro* and enhanced inhibition of glioma growth in a mouse model *in vivo* (28). The present study found that CD19pbCAR T cells had similar antitumor activity and weak activation of IL-6, simplified the steps required to prepare the vectors and reduced the cost of preparation of CAR T cells (data not shown). These findings suggest the need for clinical studies testing the use of PB in CAR T cell immunotherapy.

## Conclusion

This study showed that *piggyBac*-based CD19pbCAR T cells demonstrated high proliferation ability and strong cytotoxic activity in eliminating Raji cells *in vitro* and Raji cell-induced xenograft tumors *in vivo*. Upon injection, CD19pbCAR T cells localize to mesenteric lymph nodes, bone marrow of the femur, spleen, kidneys, lungs, and CD19-rich areas. Compared with CD19lvCAR T cells, CD19pbCAR T cells exhibited increased levels of INF-γ, TNF-α, and IL-4 and numbers of Tcm cells. The slight effects of these cells on IL-6 and IL-10 levels may reduce the risk of CAR T-cell-associated side-effects. Taken together, these results indicate that CD19pbCAR may be a valuable vector for cell drug-specific treatment of CD19^+^ B-cell malignancies.

## Data Availability Statement

The original contributions presented in the study are included in the article/**Supplementary Material**. Further inquiries can be directed to the corresponding authors.

## Ethics Statement

The animal study was reviewed and approved by Ethics Committee of Beijing IDMO Co., Ltd.

## Author Contributions

ZCL, YS, and QQ designed the study. XL, TL, HG, XT, JC, ZC, FX, and LL performed experiments and collected data. ZCL and ZL analyzed and interpreted data. Statistical analysis was done by LL and XT. ZL wrote the manuscript. ZL, YS, and QQ revised the manuscript. QQ funded the experiments. All authors contributed to the article and approved the submission version.

## Funding

This study was funded by Shanghai Cell Therapy Corporation (BZ019-Phase I) and the National Key R&D Program of China (2019YFC316202, 2019YFC1316205). The funders were not involved in the study design, collection, analysis, interpretation of data, the writing of this article or the decision to submit it for publication.

## Conflict of Interest

All authors are employed by Shanghai Cell Therapy Group Corporation.

## Publisher’s Note

All claims expressed in this article are solely those of the authors and do not necessarily represent those of their affiliated organizations, or those of the publisher, the editors and the reviewers. Any product that may be evaluated in this article, or claim that may be made by its manufacturer, is not guaranteed or endorsed by the publisher.

## Supplementary Material

Supplementary Figure 1 Expression of exhaustion markers in CD19pbCAR T and CD19lvCAR T cells. T cells from two donors were used to generate CAR T cells, and their expression of the exhaustion markers PD-1, LAG3, and TIM3 were evaluated by flow cytometry on day 13.
