# Supplementary figures and images for: Evaluation of Nonviral piggyBac and lentiviral Vector in Functions of CD19chimeric Antigen Receptor T Cells and Their Antitumor Activity for CD19+ Tumor Cells
Source: Front Immunol. 2022 Jan 10;12:802705. doi: 10.3389/fimmu.2021.802705 (PMC8784881; doi:10.3389/fimmu.2021.802705)

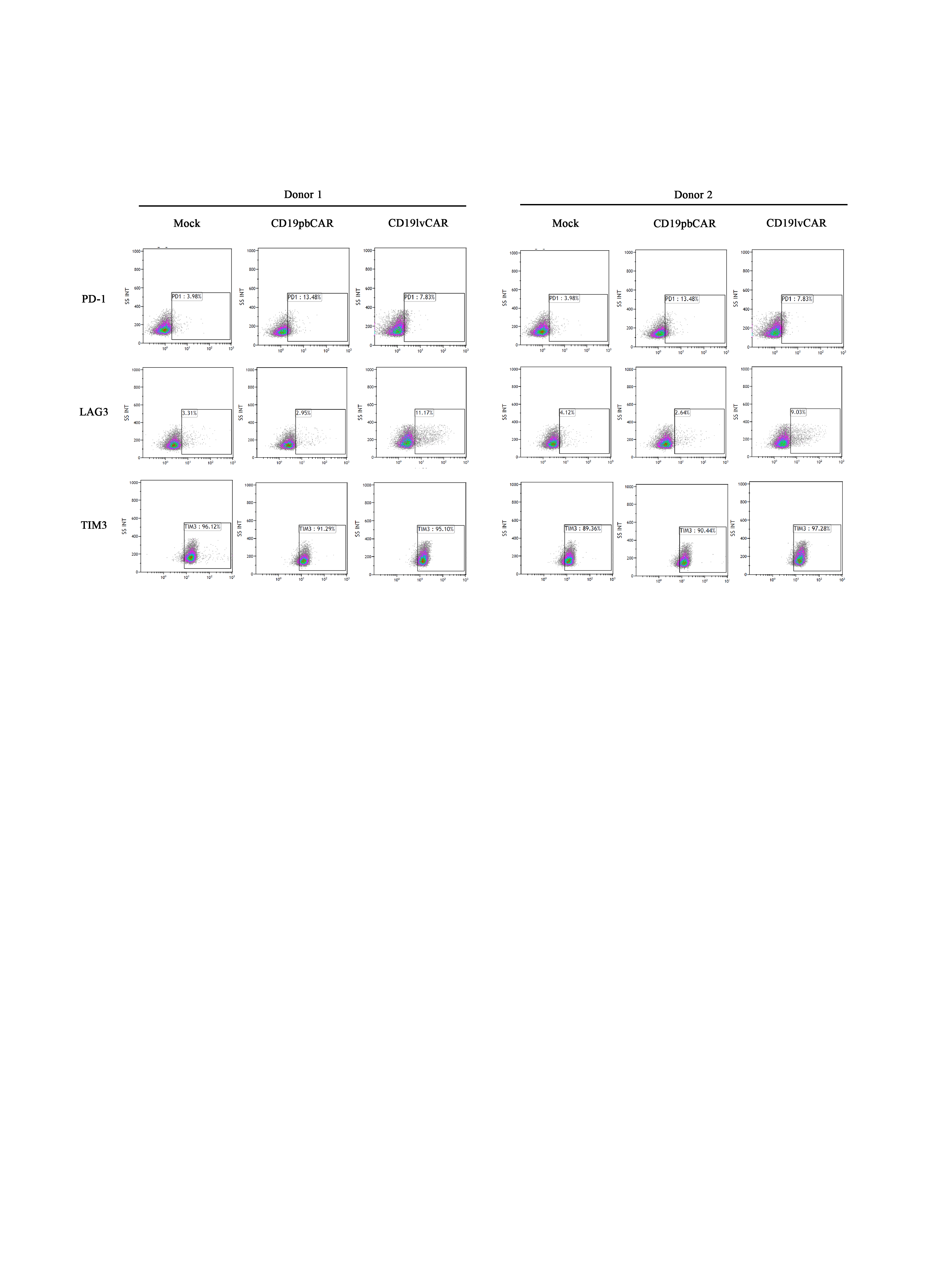

Supplement: Supplementary Figure 1 — Expression of exhaustion markers in CD19pbCAR T and CD19lvCAR T cells. T cells from two donors were used to generate CAR T cells, and their expression of the exhaustion markers PD-1, LAG3, and TIM3 were evaluated by flow cytometry on day 13. [file Image_1.tif]
